# Alcohol-Associated Hepatitis: Translating Pathophysiology into Targeted Clinical Trials

**DOI:** 10.1007/s11901-025-00703-9

**Published:** 2025-10-27

**Authors:** Jing Ma, Hui Gao, Ge Zeng, Nazmul Huda, Yanchao Jiang, Themis Thoudam, Zhihong Yang, Suthat Liangpunsakul

**Affiliations:** 1 Division of Gastroenterology and Hepatology, Department of Medicine, Indiana University School of Medicine, 702 Rotary Circle, Indianapolis, IN 46202 USA; 2 Department of Infectious Diseases, Nanfang Hospital, Southern Medical University, Guangzhou, China; 3 Department of Biochemistry and Molecular Biology, Indiana University School of Medicine, Indianapolis, IN USA; 4 Roudebush Veterans Administration Medical Center, Indianapolis, IN USA

**Keywords:** Alcohol-associated hepatitis, Clinical trial, Therapeutic targets

## Abstract

**Purpose of Review:**

Alcohol-associated hepatitis (AH) is a severe manifestation of alcohol-associated liver disease with high short-term mortality and limited treatment options. This review synthesizes mechanistic insights into AH pathogenesis and evaluates both failed and emerging clinical trials to guide targeted therapeutic development.

**Recent Findings:**

AH arises from intertwined mechanisms including hepatotoxicity, oxidative stress, inflammation, impaired regeneration, and gut-liver axis disruption. Trials targeting inflammatory cytokines or apoptosis pathways have not demonstrated survival benefit and raised safety concerns. Current investigations emphasize therapies that mitigate oxidative stress, enhance hepatocyte regeneration, and restore gut-liver integrity. Novel agents such as interleukin-22 (IL-22), granulocyte colony-stimulating factor (G-CSF), probiotics, fecal microbiota transplantation (FMT), larsucosterol, and farnesoid X receptor (FXR) agonists are under evaluation.

**Summary:**

Although no effective pharmacologic therapy is yet available, advances in understanding AH biology provide a framework for mechanism-based strategies. Integrating hepatology with addiction medicine and incorporating stratified trial designs will be essential to advance effective therapies.

## Introduction

Alcohol consumption remains a significant global public health challenge [1]. Excessive and prolonged use can lead to the development of alcohol use disorder (AUD), a chronic, relapsing condition characterized by compulsive alcohol intake despite adverse consequences [2]. One of the most serious complications of chronic alcohol use is alcohol-associated liver disease (ALD), which encompasses a spectrum of conditions ranging from simple steatosis to alcohol-associated hepatitis, cirrhosis, and hepatocellular carcinoma [2, 3]. ALD is a leading cause of liver-related morbidity and mortality worldwide, imposing a substantial burden on healthcare systems [4, 5]. Despite its severe clinical consequences, ALD remains underdiagnosed and inadequately treated.

Severe alcohol-associated hepatitis (SAH) represents the most aggressive form of ALD, characterized by high mortality rates exceeding 30% within 28 days for patients unresponsive to corticosteroids [6, 7]. SAH represents a critical unmet medical need, and despite decades of research, corticosteroids remain the only pharmacological therapy, offering only modest short-term survival benefits [8, 9]. The pathogenesis of AH is complex and involves multiple intertwined mechanisms that collectively contribute to hepatocellular injury and inflammation, and crucially, an impaired capacity for liver regeneration [10, 11]. These include direct hepatotoxicity from alcohol metabolites like acetaldehyde and reactive oxygen species, leading to cellular damage [12]. Furthermore, gut dysbiosis and increased intestinal permeability are crucial, allowing bacterial products like endotoxins to translocate from the gut to the liver, activating inflammation and immune cell activation via Kupffer cells and other immune cells [10, 13–15]. This triggers a robust inflammatory cascade with the release of pro-inflammatory cytokines such as tumor necrosis factor (TNF-α) and interleukin-6 (IL-6), driving hepatocellular damage and liver failure [10, 15]. Importantly, chronic alcohol exposure and the ongoing inflammatory milieu significantly impair the liver’s remarkable regenerative capacity, hindering the ability of hepatocytes to proliferate and repair the extensive damage, which is a major factor in disease progression and poor outcomes [11, 16]. Emerging evidence also highlights the role of genetic and epigenetic factors in influencing individual susceptibility and disease severity [17–19]. Each of these distinct pathways, metabolic dysfunction, gut-liver axis disruption, inflammation, impaired regeneration, and genetic predisposition, represents a potential target for therapeutic intervention.

**Fig. 1:**
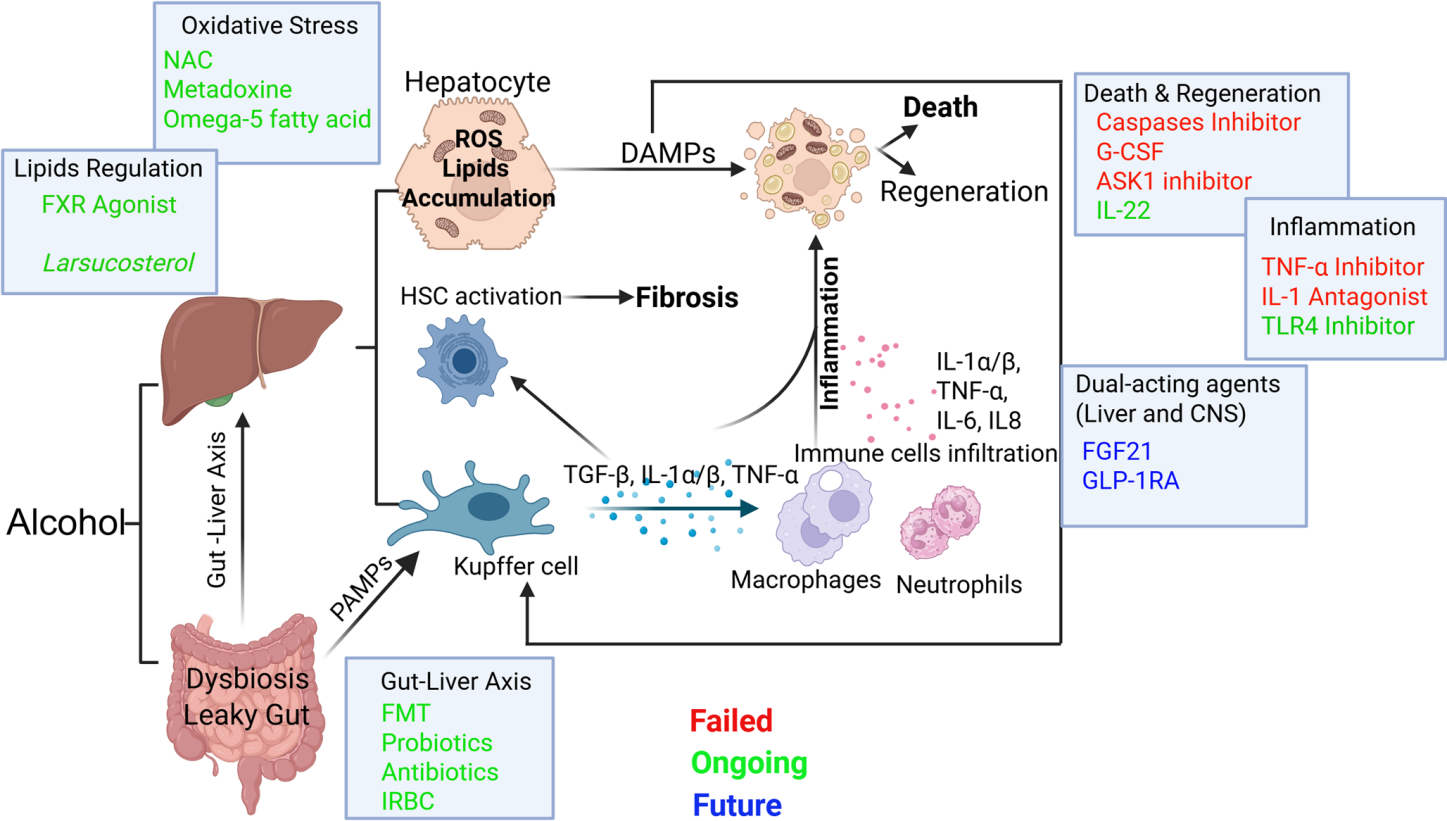
Pathophysiology of alcohol-associated hepatitis and therapeutic targets in clinical trials. Chronic alcohol consumption induces liver injury via multiple interrelated mechanisms. First, alcohol metabolism in hepatocytes generates acetaldehyde, contributing to oxidative stress, mitochondrial dysfunction, and hepatocyte apoptosis. Second, alcohol disrupts intestinal barrier integrity, disturb the microbial homeostasis, leading to increased permeability and translocation of PAMPs. PAMPs activate hepatic macrophages to produce pro-inflammatory cytokines such as IL-1β. The inflammatory cascade is further amplified by recruited monocytes derived macrophages, neutrophils and further activated hepatic stellate cells (HSCs), and liver sinusoidal endothelial cells, promoting portal fibrosis development. Past and ongoing clinical trials have evaluated novel therapies targeting critical nodes in the pathogenic cascade of AH: *Oxidative stress*: caspase inhibitor, NAC, metadoxine and Omega-5 fatty acid; *Apoptosis and regeneration*: ASK1 inhibitor, IL-22 and G-CSF.* Inflammatory signaling*: IL-1 receptor antagonist, TNF-α inhibitor and TLR4 inhibitor; *Gut–liver axis*: antibiotics, probiotics FMT and IRBC; other promising targets: FXR agonist and Larsucosterol. Future studies continue to build on these insights to refine and expand therapeutic strategies: FGF21 and GLP-1RA

This review aims to synthesize current preclinical insights and clinical trial data, critically examining both successful and, importantly, failed clinical trials to provide insights into the challenges and pitfalls encountered in drug development. By integrating this comprehensive understanding, the review will offer a roadmap for the future development of targeted therapies for AH (Fig. 1).

### Failed Therapeutic Trials in Alcohol-Associated Hepatitis

#### IL-1 Receptor Antagonist

Interleukin-1 (IL-1) is a family of proinflammatory cytokines that plays a central role in innate immune responses [20, 21]. Among its members, IL-1α and IL-1β are particularly implicated in liver inflammation [22]. Both cytokines signal through the interleukin-1 receptor type 1 (IL-1R1), activating the NF-κB and MAPK pathways and inducing the production of other proinflammatory mediators [22]. In ALD, IL-1 signaling contributes to hepatocellular injury, neutrophil recruitment, inflammasome activation, and systemic inflammation, particularly in severe AH [22, 23]. Preclinical models of ALD have shown upregulation of IL-1β in the liver, and pharmacological blockade of IL-1 signaling has been associated with reduced liver injury, steatosis, and inflammation [24]. These findings supported the rationale for targeting the IL-1 pathway in clinical trials.

Anakinra, a recombinant IL-1 receptor antagonist that inhibits both IL-1α and IL-1β, has been investigated in AH as both monotherapy and in combination with other agents. A pilot randomized controlled trial compared a combination of anakinra, pentoxifylline, and zinc to standard care with methylprednisolone in patients with severe AH [25]. However, the study lacked sufficient statistical power to draw definitive conclusions [25]. Based on these preliminary data, a phase IIb, double-blind, multicenter randomized trial was conducted to evaluate the safety and efficacy of anakinra plus zinc compared to prednisone [26]. This trial was terminated early after a pre-specified interim analysis demonstrated that prednisone significantly improved 90-day overall survival compared to anakinra group [26]. Another IL-1 pathway inhibitor, canakinumab, a monoclonal antibody targeting IL-1β, was also evaluated but failed to demonstrate clinical efficacy [27]. Taken together, despite strong preclinical rationale and early-phase promise, IL-1 blockade has not translated into meaningful clinical benefit in the treatment of severe AH.

#### TNF-α Inhibitors

TNF-α have been consistently observed increased in both animal models and patients with ALD [28]. Dysregulated TNF-α signaling is believed to contribute to the metabolic disturbances and hepatocellular injury associated with ALD [28, 29]. Given its central role in disease pathogenesis, TNF-α has been evaluated as a therapeutic target in ALD. However, clinical trials targeting TNF-α have produced largely negative outcomes. Pentoxifylline, an inhibitor of TNF-α synthesis, was initially reported to reduce short-term mortality in patients with AH, primarily by lowering the risk of hepatorenal syndrome [30]. However, the large-scale STOPAH trial failed to demonstrate a significant survival benefit of pentoxifylline compared to placebo [31]. Etanercept, a soluble TNF receptor fusion protein that binds and neutralizes circulating TNF-α, was assessed in a randomized controlled trial involving patients with moderate-to-severe AH [32]. Although TNF-α levels were reduced, patients treated with etanercept experienced a higher rate of infections and increased 6-month mortality compared to placebo, leading to early trial termination [32]. Infliximab, a monoclonal antibody that neutralizes both soluble TNF-α homotrimers and membrane-bound TNF-α precursors [33], was evaluated in a double-blind, randomized controlled trial. Within two months, high mortality rate occurred in the infliximab group compared to that in the placebo group, prompting early termination [34]. Additionally, the incidence of severe infections was higher in the infliximab group [34]. Another study assessing a single dose of infliximab showed some improvements in clinical severity and short-term survival, but infection remained a significant concern [35]. Due to these safety issues and lack of demonstrated efficacy, anti–TNF-α therapies are no longer recommended for the treatment of AH.

#### Caspases Inhibitor

Caspases, cysteine proteases involved in programmed cell death and inflammasome activation, have been identified as potential therapeutic targets in ALD due to their roles in both hepatocyte apoptosis and the maturation of proinflammatory cytokines [36]. Emricasan (IDN-6556), an orally bioavailable, irreversible pan-caspase inhibitor, has demonstrated therapeutic potential in patients with cirrhosis [37]. In a randomized clinical trial that included 38% of participants with alcohol-associated cirrhosis, a 3-month course of emricasan significantly improved liver function compared to placebo [37]. Despite these encouraging results in patients with cirrhosis, a clinical trial evaluating emricasan in patients with severe AH was terminated early due to pharmacokinetic limitations and issues with drug availability in critically ill patients with advanced liver failure (NCT01912404 [38]).

#### Selonsertib

Selonsertib is an investigational small-molecule inhibitor of apoptosis signal-regulating kinase 1 (ASK1). In preclinical models of ALD, selonsertib has shown potential in reducing hepatic inflammation and oxidative stress [39]. However, in a randomized clinical trial evaluating its efficacy in patients with AH, selonsertib did not demonstrate a survival benefit (NCT02854631 [40]).

### Current and Emerging Treatments for Alcohol-Associated Hepatitis: A Mechanistic Perspective

Recent advances in elucidating the complex pathophysiology of AH have led to the development of targeted therapeutic strategies. A growing number of novel agents have shown promising results and are currently under investigation in ongoing trials. Table 1 summarizes active clinical trials investigating these mechanistic approaches in the treatment of ALD.

### Drugs Targeting Oxidative Stress Pathways

Oxidative stress is a key pathogenic mechanism in ALD [10, 41]. Chronic alcohol consumption increases the generation of reactive oxygen species (ROS) in hepatocytes, primarily through alcohol metabolism via alcohol dehydrogenase, cytochrome P450 2E1, and mitochondrial respiratory chain dysfunction [12, 42]. Excessive ROS production leads to lipid peroxidation, mitochondrial injury, and the activation of pro-inflammatory and fibrogenic pathways [12, 41–43]. Despite the mechanistic rationale, antioxidant therapies have not demonstrated significant clinical benefit in the treatment of AH [44–48]. Nevertheless, several emerging therapeutic agents targeting oxidative stress are currently under investigation.

#### N-acetylcysteine

N-acetylcysteine (NAC) is a potent antioxidant that replenishes intracellular glutathione [49]. In a double-blind, randomized controlled trial, the addition of NAC to prednisolone reduced 1-month mortality compared to prednisolone alone in patients with severe AH [50]. However, this benefit did not persist beyond the short term, with no improvement in long-term survival [50]. A Phase 3 clinical trial is currently underway to investigate the mechanisms by which NAC may reduce infection susceptibility in patients with AH, including potential enhancement of monocyte oxidative burst activity. Secondary outcomes of the study include assessments of infection rates and all-cause mortality (NCT03069300 [51]).

#### Metadoxine

Metadoxine (pyridoxol L-2-pyrrolidone-5-carboxylate, MTD) is a compound combining pyridoxine and pyrrolidone carboxylate. It has been reported to increase hepatic adenosine triphosphate concentrations, restore reduced glutathione levels, and protect against the inhibition of tryptophan pyrrolase activity [52, 53]. Additionally, MTD enhances the plasma clearance of ethanol and acetaldehyde, thereby reducing hepatic and systemic exposure to these toxic metabolites [54, 55]. In a randomized open-label clinical trial, patients receiving MTD plus prednisone demonstrated significantly higher 3- and 6-month survival rates compared to those receiving prednisone or pentoxifylline monotherapy [56]. While these findings are encouraging, the therapeutic efficacy of MTD remains to be validated in larger, rigorously designed, double-blind, placebo-controlled trials.

#### Omega-5 Fatty Acid (punicic Acid)

Omega-5 fatty acid is a potent antioxidant and a peroxisome proliferator-activated receptor gamma agonist that reduces lipid peroxidation and restores antioxidant enzymes, including superoxide dismutase, catalase, and glutathione peroxidase [57, 58]. It also exerts strong anti-inflammatory effects by inhibiting TNFα-induced priming of nicotinamide adenine dinucleotide phosphate (NADPH) oxidase [59]. A double-blind clinical trial demonstrated that oral administration of Omega-5, in combination with prednisone, significantly reduced systemic lipid oxidative stress [60] (NCT03732586 [61]).

### Drugs Targeting Inflammatory Signaling

Inflammation is a central driver of AH [62]. As a result, therapies targeting inflammation have become a major focus in recent clinical trials. TAK‑242 is a small-molecule inhibitor of toll-like receptor 4 (TLR4), a key mediator of innate immune activation [63]. TLR4 is activated by gut-derived endotoxins such as lipopolysaccharide (LPS), leading to exaggerated inflammatory responses, hepatocyte injury, and multi-organ failure [28, 64]. In a mouse model of LPS-induced acute-on-chronic liver failure (ACLF), the addition of TAK-242 to granulocyte colony-stimulating factor (G-CSF) completely prevented mortality and significantly reduced hepatocellular injury, macrophage infiltration, and inflammation [65]. TAK-242 is currently being evaluated in a randomized controlled trial in patients with acute decompensation of alcohol-associated cirrhosis (ALC) due to AH (NCT04620148 [66]).

### Drugs Targeting Cell Survival and Liver Regeneration

Hepatocyte apoptosis is a central contributor to liver injury in AH, driving inflammation, fibrosis, and impaired regeneration [67, 68]. Excessive cell death can overwhelm the liver’s intrinsic regenerative capacity, resulting in poor clinical outcomes [11, 67–69]. Preclinical studies have demonstrated that therapeutic strategies aimed at inhibiting apoptosis while simultaneously promoting hepatic regeneration can enhance hepatocyte survival and support functional recovery [69]. These findings highlight the rationale for clinical trials targeting these pathways.

#### Interleukin-22 (IL-22)

IL-22, a member of the IL-10 cytokine family, plays a dual role in mediating inflammation and promoting tissue protection in various disease contexts [70, 71]. IL-22 is primarily produced by innate lymphoid cells, Th17 cells, and γδ T cells, and signals through a heterodimeric receptor complex composed of IL-22R1 and IL-10R2 [72, 73]. This receptor is predominantly expressed on non-hematopoietic cells, including hepatocytes, epithelial cells, and keratinocytes [72, 73]. In the liver, IL-22 exerts hepatoprotective, anti-apoptotic, anti-steatotic, and pro-regenerative effects, primarily via activation of the signal transducer and activator of transcription 3 (STAT3) signaling pathway [74]. Unlike classical pro-inflammatory cytokines such as TNF-α or IL-1β, IL-22 does not directly activate immune cells, reducing the risk of inducing systemic inflammation [70]. Among IL-22–based therapies, the most extensively studied candidate is F-652, a recombinant fusion protein consisting of human IL-22 linked to an immunoglobulin G subclass 2 – fragment crystallizable region (IgG2-Fc) fragment. In a Phase 2 clinical trial, F-652 was administered to patients with moderate to severe AH, demonstrating a favorable safety profile and tolerability, along with improvements in liver function and short-term survival outcomes [75]. The treatment was associated with a decrease in inflammatory cytokines and increases in markers of hepatic regeneration [75]. Building on these findings, a prospective, multicenter, NIAAA-supported trial with severe AH is currently underway to further evaluate F-652. This trial uses a sequential randomization design to assess whether integrating alcohol use disorder interventions with severe AH therapies improves a composite endpoint of alcohol- and liver-related events at 6 months compared to usual care (NCT07060638 [76]).

#### Granulocyte Colony-Stimulating Factor (G-CSF)

G-CSF is a hematopoietic growth factor that promotes the proliferation, differentiation, and mobilization of granulocyte precursors from the bone marrow [77, 78]. Beyond its hematologic functions, G-CSF exerts immunomodulatory effects and mobilizes both hematopoietic and hepatic progenitor cells, which may home to the liver and contribute to tissue repair and regeneration [79–81]. In preclinical models of liver injury, G-CSF has demonstrated hepatoprotective and regenerative effects [82, 83], making it a biologically plausible therapeutic candidate for patients with severe AH. Early clinical trials, particularly in South Asian populations, reported encouraging results. In a randomized, double-blind, single-center trial, G-CSF significantly improved 90-day survival and reduced model for end-stage liver disease (MELD) and Maddrey’s discriminant scores in patients with steroid-nonresponsive severe AH; the incidence of infections was also lower in the G-CSF group compared to placebo [84]. However, results from trials conducted in Western populations have been less favorable. A Phase II, multicenter, open-label randomized trial conducted in India evaluating pegfilgrastim (recombinant G-CSF) showed no survival benefit at 90 days [85]. Similarly, the GRAFT trial, a European multicenter randomized study, failed to demonstrate a significant survival benefit of G-CSF compared to standard of care in patients with ACLF, of whom 55% in the G-CSF group had alcohol-associated liver disease [86]. These discrepancies may be attributed to heterogeneity in disease severity, timing and duration of G-CSF administration, differences in baseline immune function, or underlying genetic and environmental factors. Despite these mixed results, G-CSF remains a mechanistically attractive therapy for AH due to its dual regenerative and immunomodulatory properties. Future stratified trials are warranted to better define its therapeutic role and to identify patient subgroups most likely to benefit.

### Drugs Targeting Gut-liver Axis

Chronic alcohol consumption disrupts the gut microbiota, leading to an overgrowth of pathogenic bacteria and increased production of bacterial endotoxins, particularly LPS [87, 88]. Alcohol also impairs intestinal barrier integrity by downregulating tight junction proteins, thereby facilitating the translocation of microbial products into the portal circulation [87–89]. These pathogen-associated molecular patterns (PAMPs), such as LPS, activate hepatic immune cells, especially Kupffer cells, initiating inflammatory cascades that exacerbate liver injury [88, 90]. Given the central role of gut dysbiosis and microbial translocation in the pathogenesis of AH, several microbiome-targeted strategies have been explored.

#### Probiotics and Synbiotics

Probiotics such as *Lactobacillus rhamnosus* GG (LGG) have demonstrated therapeutic potential in preclinical models and early clinical studies. A prospective, randomized clinical trial evaluated probiotic supplementation in ALD [91]. In this study, 32 patients were randomized to receive oral Bifidobacterium bifidum and Lactobacillus plantarum 8PA3, while 34 patients received standard therapy consisting of abstinence and vitamin supplementation [91]. Thirteen patients in each group had laboratory findings consistent with AH [91]. Short-term supplementation significantly restored gut microbial composition and improved liver enzyme levels compared to standard therapy [91]. A multicenter, randomized, double-blind study demonstrated that a 7-day course of probiotic treatment with cultured Lactobacillus subtilis and Streptococcus faecium resulted in higher serum albumin levels, reduced TNF-α and serum LPS concentrations, and decreased Escherichia coli counts compared to placebo in patients with alcohol-associated hepatitis [92]. In a separate randomized, double-blind, placebo-controlled trial, oral administration of Lactobacillus rhamnosus R0011 and Lactobacillus helveticus R0052 was associated with restoration of gut microbial balance and improvements in clinical parameters, including lower Child–Pugh scores, alanine aminotransferase (ALT), and gamma-glutamyl transferase (GGT) levels [93]. Further evidence comes from a randomized trial in patients with moderate AH, where LGG supplementation resulted in significant improvements in MELD score and aspartate aminotransferase (AST)/ALT ratio at 1 month. Notably, patients receiving LGG also exhibited a significant reduction in alcohol consumption at 6 months compared to placebo [94]. Ongoing studies continue to evaluate the potential of probiotic therapy in alcohol-associated hepatitis. A randomized, parallel-group trial (NCT05178069 [95]) is assessing the effects of a 6-month Lactobacillus rhamnosus GG (LGG) regimen on liver injury and reduction of heavy drinking in patients with alcohol use disorder and moderate AH. The estimated completion date is February 2027. Another randomized controlled trial (NCT03863730 [96]) is investigating the impact of probiotics on alcohol-related gut dysbiosis and disease progression, with an expected completion in February 2031.

#### Fecal Microbiota Transplantation (FMT)

FMT is a promising intervention to restore healthy intestinal flora in AH. In an initial pilot study, FMT was administered to steroid-ineligible severe AH patients, demonstrating feasibility and potential clinical benefit [97]. Subsequently, an open-label trial compared FMT with corticosteroids, pentoxifylline, and nutritional therapy in 51 male patients with severe AH. The FMT group showed significantly higher survival rates at both one and three months, with three out of four patients surviving, compared to the other treatment groups [98]. A follow-up study comparing 35 FMT-treated patients to 26 standard-of-care controls reported that FMT was associated with reduced rates of ascites, hepatic encephalopathy, infections, hospitalizations, and alcohol relapse, along with a trend toward improved 3-year survival [99]. Similarly, another open-label study observed improved survival at 28 and 90 days following fecal microbiota transplantation (FMT). Among survivors, hepatic encephalopathy resolved in all patients who received FMT compared to 57% in the standard-of-care group, while ascites resolved in all FMT patients versus 40% of those receiving standard care [100]. Moreover, a European prospective study demonstrated that FMT improved 30-day mortality, particularly in patients with MELD ≤ 30, Maddrey’s discriminant function ≤ 90, and ACLF grade < 2 [101]. A recent systematic review including 371 patients further corroborated these results, showing that FMT nearly tripled short-term survival at 1 and 3 months, although benefits beyond 6 months were not sustained [102]. A randomized controlled trial (NCT06496191 [103]) is currently underway to rigorously evaluate the efficacy and safety of FMT in patients with alcohol-related cirrhosis.

#### Antibiotics

Patients with severe AH or advanced cirrhosis are highly susceptible to bacterial infections due to increased intestinal permeability, bacterial translocation, and immune dysfunction [104]. These infections are a major contributor to multi-organ failure and mortality [104, 105]. In this context, antibiotic therapy has been investigated in several clinical trials. Rifaximin, a non-absorbable antibiotic widely used in cirrhotic patients, has also been evaluated in the setting of AH [106]. In a pilot study, rifaximin reduced the number of infections per patient and decreased liver-related complications compared to controls [106]. The incidence of infection-related ACLD was lower, and a reduction in mortality was observed, although this difference did not reach statistical significance [106]. However, findings have not been uniformly positive. One randomized controlled trial found that rifaximin did not significantly alter markers of inflammation or metabolic function in patients with AH [107]. Another trial evaluating the addition of amoxicillin-clavulanate to prednisolone in patients with SAH reported no improvement in 2-month survival compared to prednisolone alone [108]. A recent systematic review and meta-analysis evaluating prophylactic antibiotic use in patients with AH receiving corticosteroids found that, although antibiotics reduced the incidence of infections during treatment, this did not translate into a significant improvement in overall survival, highlighting the need for individualized infection prevention strategies rather than routine universal prophylaxis [109].

#### Immunoglobulin-rich Bovine Colostrum (IRBC)

Bovine colostrum, the nutrient-rich fluid secreted by cows during the first few days postpartum, contains high concentrations of immunoglobulins (primarily IgG), antimicrobial peptides, lactoferrin, growth factors, and other bioactive components that support mucosal immunity and gut barrier function [110]. IRBC, derived from hyperimmunized cows, are enriched with broad-spectrum neutralizing antibodies targeting bacterial endotoxins and enteric pathogens [111]. In preclinical models, colostrum supplementation has been shown to reduce inflammatory responses [112, 113]. An open-label, single-arm study in patients with severe AH who were in extremis demonstrated significant improvement in Maddrey’s discriminant function score following IRBC treatment [114]. Two clinical trials have evaluated IRBC in the context of AH. One is an ongoing Phase 3, double-blind, randomized controlled trial across multiple centers (NCT02473341 [115]). The other, a completed Phase 2a, multicenter, randomized, double-blind, placebo-controlled trial, tested IMM-124E, a purified hyperimmune bovine colostrum, in patients with severe AH. The primary aim was to assess safety and proof-of-concept efficacy by measuring endotoxin levels, inflammatory biomarkers, and clinical endpoints; final results are pending (NCT01968382 [116]).

### Other Promising Therapeutic Targets

#### Larsucosterol

Larsucosterol (25-hydroxycholesterol-3-sulfate; 25HC3S or DUR-928) is an endogenous cholesterol metabolite and a member of a novel class of small-molecule epigenetic regulators that modulate nuclear receptors implicated in lipid metabolism, inflammation, and tissue regeneration [117]. The AHFIRM trial was a multinational, randomized, double-blind, placebo-controlled Phase 2b study designed to evaluate the safety and efficacy of larsucosterol, a novel epigenetic modulator, in patients with severe AH [118]. The study enrolled over 300 participants across the U.S., Europe, the U.K., and Australia, who were randomized to receive either larsucosterol (30 mg or 90 mg) or placebo, with all groups receiving standard supportive care [118]. The primary endpoint was the incidence of death or liver transplantation by 90 days [118]. Although the trial did not achieve its primary endpoint in the overall study population, a pre-specified subgroup analysis demonstrated a statistically significant reduction in 90-day mortality among participants in the United States, but not in Europe, with both larsucosterol doses associated with greater than 50% lower mortality compared to placebo [118]. The difference appears to be driven largely by earlier treatment initiation in the U.S., where patients generally received larsucosterol sooner after hospitalization, while delays in treatment at European sites likely blunted the drug’s effectiveness. Additional factors, such as variations in standard of care, trial logistics, and baseline patient characteristics between regions, may also have contributed to the discrepancy in outcomes [118]. Larsucosterol was well tolerated, with no serious adverse events attributed to the drug and fewer treatment-emergent adverse events observed in the treatment arms [118]. Based on these encouraging findings, particularly in the U.S. population, a Phase 3 trial is planned to further evaluate its potential as a disease-modifying therapy for severe AH.

#### Farnesoid X Receptor (FXR) Agonist

Dysregulation of bile acid metabolism and nuclear receptor signaling contributes to hepatocellular injury and disrupted lipid homeostasis [119]. Among the nuclear receptors regulating bile acid pathways, the FXR has emerged as a promising therapeutic target [120]. An earlier Phase 2 trial of obeticholic acid, a first-generation FXR agonist, in patients with moderate to severe AH was terminated prematurely (NCT02039219 [121]). A next-generation FXR agonist, INT-787, is currently under investigation in a Phase 2a clinical trial for the treatment of severe AH (FRESH study; NCT05639543 [122]). This randomized, double-blind, placebo-controlled dose-escalation study is evaluating the safety, tolerability, pharmacokinetics, pharmacodynamics, and preliminary efficacy of INT-787 in hospitalized severe AH patients.

**Table 1:** Ongoing clinical trial

Trial title	Intervention	Outcome Measure	Study design	Phase	study population	Study status	Completion date (estimated)	NCT Number
TAK-242 in Patients With Acute Alcoholic Hepatitis	TAK-242 (TLR 4 inhibitor)	Change in CLIF-C ACLF score from baseline to Day 8	Randomized, Parallel Assignment	Phase II	Acute alcoholic hepatitis	pending	12/1/2022	NCT04620148
Utility of the Use of N-acetylcysteine Associated With Conventional Treatment in Patients With Severe Acute Alcoholic Hepatitis (Maddrey> 32)	NAC	Number of Participants with all-cause mortality at 6 months	Randomized, controlled, multicenter, parallel and open trial	N/A	Acute alcoholic hepatitis	unknown	7/1/2023	NCT05294744
Fecal Microbiota Therapy in Steroid Ineligible Alcoholic Hepatitis	FMT	Mortality at 3 months, Liver transplant free survival	A prospective, randomized controlled trial	N/A	Steroid ineligible severe alcoholic hepatitis	unknown	3/31/2024	NCT05285592
Study of Decompensated Alcoholic Cirrhosis Treatment by Stem Cells	conventional therapy plus UCMSCs treatment	SIAE after the administration of UCMSCs, survival rate	Non-Randomized, Sequential Assignment	Phase I	Alcoholic liver cirrhosis	unknown	12/25/2024	NCT05155657
Role of Extended Low Dose Prednisolone in Achieving Clinical and Biochemical Remission in Steroid Responsive Severe Alcoholic Hepatitis	Prednisolone 10 mg	The proportion of steroid responsive SAH patients achieving remission by extended low dose Prednisolone (10mg/day) till day 90 in comparison to SMT	Randomized, Parallel Assignment	N/A	Alcohol-associated hepatitis	Not yet recruiting	2/27/2025	NCT06155760
N-ACetylcysteine to Reduce Infection and Mortality for Alcoholic Hepatitis (NACAH)	NAC	Improvement in monocyte oxidative burst	Randomised controlled trial, open label	Phase III	Alcoholic hepatitis	unknown	6/1/2025	NCT03069300
Safety Evaluation of Fecal Microbiota Transplantation in Severe Alcoholic Hepatitis	FMT (PRIM-DJ2727)	Survival, change in gut microbiome	Double Blinded, Randomized, Placebo-Controlled Study	Phase I, II	Severe alcoholic hepatitis	Recruiting	6/30/2025	NCT05006430
FMT for Alcohol Use Disorder in Cirrhotics	FMT	To assess the efficacy of FMT in decreasing lapse, relapses and maintaining alcohol abstinence in AUD in patients with cirrhosis	Open label, parallel group, randomized, controlled study	N/A	Alcohol related cirrhosis	Recruiting	6/30/2025	NCT06496191
Gut-Liver Axis Modulation With IgG-Enriched Immunotherapy in Severe Alcohol-Associated Hepatitis (BC-SAAH)	Bovine Colostrum	Survival at 3 month	Randomized, Parallel Assignment	Phase III	Severe alcoholic hepatitis	Active, not recruting	9/1/2025	NCT02473341
SAMe Trial for Patients with Alcoholic Cirrhosis	SAMe 400 mg tablet	Effect on all-cause mortality	Randomized, Parallel Assignment	Phase II	Alcoholic cirrhosis	Recruiting	9/1/2025	NCT04250259
Alcoholic Liver Disease and the Gut Microbiome	VSL #3 (Probiotic)	Sex differences within ALD	Randomized placebo controlled trial	N/A	AUD and ALD	Active, not recruting	9/1/2025	NCT05007470
Nutritional Interventions in Patients With Alcohol-associated Hepatitis	Nutritional Interventions	Transplant free survival (30 days)	Randomized, Parallel Assignment	N/A	Acute severe AH	Not yet recruiting	12/31/2025	NCT06131177
HA35 Moderate Alcoholic Hepatitis (AH) Study	HA35 (Sodium Hyaluronate)	Percent change of skeletal muscle mass	Randomized, Parallel Assignment	Phase I	Severe alcoholic hepatitis	Recruiting	10/1/2026	NCT05018481
MRG-001 in Patients With Alcoholic Hepatitis	MRG-001	Assess the dose related safety, Pharmacokinetics, and Pharmacodynamics of MRG-001 in patients with severe alcoholic hepatitis	Open-Label, Dose-Escalation	Phase II	Alcoholic hepatitis	Not yet recruiting	12/1/2026	NCT06307522
LGG Supplementation in Patients With AUD and ALD (AUD+ALD)	Probiotic	No heavy drinking days, WHO 1/2 -level reductionliver related and clinical markers	Randomized, Parallel Assignment	Phase II	AUD and ALD	Recruiting	2/28/2027	NCT05178069
FXR Effect on Severe Alcohol-Associated Hepatitis (FRESH) Study (FRESH)	INT-787 (FXR agonist)	Lille model response based on Lille score	Randomized, Single Group Assignment	Phase II	Severe alcoholic hepatitis	Recruiting	4/1/2027	NCT05639543
Integrated Therapies for Alcohol Use in Alcohol-associated Liver Disease (ITAALD) Trial (ITAALD)	F-652 (IL-22)	Death, liver transplant, ascite, hepatic encephalopathy, portal hypertensive bleeding, liver-related hospital admission, Increase in MELD score > 5 points, return to drinking	Randomized, Sequential Assignment	Phase II	Alcohol associated hepatitis	Not yet recruiting	12/1/2029	NCT07060638
Profermin®: Prevention of Progression in Alcoholic Liver Disease by ModulatingDysbiotic Microbiota (SYN-ALD)	Profermin Plus, FSMP, probiotics	Hepatic stellate cell activity	Randomized Controlled Clinical Trial	N/A	Compensated advanced chronic alcohol-related liver disease	Active, not recruting	2/1/2031	NCT03863730

## Future Perspective and Conclusion

Despite extensive research into the pathophysiology of AH, effective treatments remain limited. Prednisolone remains the guideline-recommended treatment for severe AH (typically 40 mg daily), although the optimal dosing strategy has been debated. The recent STASH trial directly compared a fixed-dose regimen (40 mg/day for 4 weeks) with a rapid taper (40 mg/day with weekly 10 mg reductions over 4 weeks) [123]. The tapered regimen was associated with a significantly lower incidence of infections, including fewer microbiologically confirmed infections, without differences in mortality, acute kidney injury, readmissions, or overall adverse events [123]. These findings suggest that a rapidly tapered steroid regimen may represent a safer alternative that mitigates infection risk without loss of efficacy, whereas standard 4-week fixed dosing may unnecessarily increase infectious complications in this high-risk population [123]. Moreover, recent advances have paved the way for mechanism-based therapeutic development. Future strategies will likely target both hepatic injury and systemic drivers of disease progression, such as inflammation and alcohol use behavior. A particularly promising approach involves dual-acting agents with hepatoprotective properties and central nervous system activity to reduce alcohol consumption. These therapies address both the liver damage characteristic of AH and the underlying AUD, a key contributor to disease progression and recurrence. Among these are fibroblast growth factor 21 (FGF21) analogs and glucagon-like peptide-1 receptor agonists (GLP-1 RAs). FGF21 is an endocrine hormone that has demonstrated protective effects against hepatic steatosis, inflammation, and fibrosis in alcoholic fatty liver disease models [124, 125]. In parallel, it modulates reward-related neural pathways, reducing alcohol preference and intake in both animal studies and early-phase clinical trials [126, 127]. Similarly, GLP-1 RAs, originally developed for the treatment of type 2 diabetes and obesity [128–130], have shown promising hepatoprotective and anti-inflammatory properties in preclinical models of alcohol-induced liver injury [131]. These preclinical findings are supported by emerging human data. A target trial emulation using a cohort of U.S. Veterans with hazardous alcohol use found that GLP-1 RA initiation was associated with a lower risk of composite liver-related outcomes and death, as well as a reduced likelihood of a positive AUDIT-C score during follow-up [132]. Furthermore, a small randomized controlled trial of semaglutide in individuals with AUD demonstrated significant reductions in alcohol intake, craving, and heavy drinking days over a nine-week period [133]. Taken together, FGF21 analogs and GLP-1 RAs represent a promising new class of dual-targeted therapies that align closely with the underlying pathophysiology of AH. Their ability to simultaneously target hepatic injury and modulate alcohol-related behavior offers a compelling and more holistic therapeutic strategy for individuals affected by both AUD and ALD.

Looking ahead, successful therapeutic innovation in AH will depend not only on targeting disease mechanisms but also on optimizing trial design, patient stratification, and integrated care models [134, 135]. Furthermore, integrating treatments for AH with behavioral and pharmacologic interventions for AUD is essential for long-term disease control. As the field moves forward, cross-disciplinary collaboration between hepatologists and addiction medicine specialists will be critical to translating mechanistic insights into effective therapies.

## Key References

- Shiffman M, et al. Larsucosterol for the Treatment of Alcohol-Associated Hepatitis. NEJM Evid. 2025 Feb;4(2): EVIDoa2400243.
  - This study is the first randomized, placebo-controlled trial of larsucosterol in patients with SAH, showing overall neutral results but suggesting potential benefit in US patient subgroup with good safety.
- John BV, et al. Association of Glucagon-Like Peptide-1 Receptor Agonists With Liver-Related Outcomes and All-Cause Mortality in Patients With Harmful Alcohol Use: A Target Trial Emulation Study. Am J Gastroenterol. 2025 Jun.
  - This pioneering target trial emulation study reveals that GLP-1 receptor agonists are associated with reduced liver-related complications and all-cause mortality in high-risk patients with harmful alcohol use.
- Mackowiak B, et al. Alcohol-associated liver disease. J Clin Invest. 2024 Feb 1;134(3): e176345.
  - This comprehensive review provides a new insight into the pathogenesis of ALD.
- Engelmann C, et al. Granulocyte-colony stimulating factor (G-CSF) to treat acute-on- chronic liver failure: A multicenter randomized trial (GRAFT study). Journal of Hepatology. 2021 Dec;75(6):1346-1354.
  - This multicenter, prospective, controlled, open-label phase II study demonstrated that G-CSF had no significant beneficial effect on patients with ACLF.
- Arab JP, et al. An Open-Label, Dose-Escalation Study to Assess the Safety and Efficacy of IL-22 Agonist F-652 in Patients with Alcohol-associated Hepatitis. Hepatology. 2020 Aug;72(2):441-453.
  - This early-phase dose-escalation study provides initial evidence supporting the safety and preliminary efficacy of the IL-22 agonist F-652 in patients with AH, highlighting its therapeutic potential.
- Han SH, et al. Effects of probiotics (cultured Lactobacillus subtilis/Streptococcus faecium) in the treatment of alcoholic hepatitis: randomized-controlled multicenter study. Eur J Gastroen Hepat. 2015 Nov;27(11):1300-6.
  - As the first clinical trial in alcohol-associated hepatitis patients, this multicenter randomized-controlled study demonstrates that probiotic therapy (Lactobacillus subtilis/Streptococcus faecium) is safe and may improve clinical outcomes, supporting its therapeutic potential.
- Taha AM, et al. Impact of fecal microbiota transplantation in severe alcoholic hepatitis: A systematic review and meta-analysis. JGH Open. 2024 Aug 19;8(8):e70007.
  - Findings from this meta-analysis highlights the potential of FMT to significantly improve short-term survival rates in SAH patients.
- Quek JWE, et al. Prophylactic antibiotics in patients with alcohol-associated hepatitis receiving steroids: A systematic review and meta-analysis. Liver Int. 2024 Sep;44(9):2469-2476.
  - The meta-analysis highlights prophylactic antibiotics reduced the risk of infection and hepatic encephalopathy in hospitalized AH patients receiving steroid therapy.
- Kulkarni AV, et al. Infections in Standard or Tapered Dose of Prednisolone for Alcohol-Associated Hepatitis: A Randomized Trial (STASH Trial). Am J Gastroenterol. 2025 Mar 13.
  - This report expanded our understanding of hormone therapy and uncovered a tapered prednisolone regimen may mitigate the frequency of infections in patients with SAH.
- Liangpunsakul, S, et al., FGF21 responses to alcohol, an insight from a comparative study in individuals with alcohol use disorder. Alcohol, 2025 Feb:122:11-13.
  - This report is the first comprehensively to compare FGF21 dynamics between AUD patients and controls during alcohol exposure, this work reveals critical dysregulation in a hormone central to metabolic health and craving.

## Data Availability

No datasets were generated or analysed during the current study.

## Abbreviations

ACLF: Acute-on-chronic liver failure
ADH: Alcohol dehydrogenase
AH: Alcohol-associated hepatitis
ALC: Alcohol-associated cirrhosis
ALD: Alcohol-associated liver disease
ALT: Alanine aminotransferase
ASK1: Apoptosis signal-regulating kinase 1
AST: Aspartate aminotransferase
AUD: Alcohol use disorder
FGF21: Fibroblast growth factor 21
FMT: Fecal microbiota transplantation
FXR: Farnesoid X receptor
G-CSF: Granulocyte colony-stimulating factor
GGT: Gamma-glutamyl transferase
IgG2-Fc: Immunoglobulin G subclass 2 – fragment crystallizable region
IL: Interleukin
IL-1R1: Interleukin-1 receptor type 1
IRBC: Immunoglobulin-rich bovine colostrum
LPS: Lipopolysaccharide
MAPK: Mitogen-activated protein kinase
MELD: Model for end-stage liver disease
MTD: Metadoxine
NAC: N-acetylcysteine
NADPH: Nicotinamide adenine dinucleotide phosphate
NF-κB: Nuclear factor kappa B
PAMPs: Pathogen-associated molecular patterns
ROS: Reactive oxygen species
STAT3: Signal transducer and activator of transcription 3
TLR4: Toll-like receptor 4
TNF-α: Tumor necrosis factor-alpha
